# Integrated analysis of *Cosmos sulphureus* stem fibers as thermally stable and eco-compatible reinforcements in biocomposites

**DOI:** 10.1038/s41598-025-23756-8

**Published:** 2025-11-13

**Authors:** Palanivendhan Murugadoss, R. Vigneswaran, Kulmani Mehar, C. G. Ramachandra, Honganur Raju Manjunath, Dhirendra Nath Thatoi, D. Dhorajiya Amitkumar, Kamakshi Priya Kumar

**Affiliations:** 1 Center for Automotive Materials, Department of Automobile Engineering, College of Engineering and Technology, SRM Institute of Science and Technology, Kattankulathur, Kanchipuram, 603203 Tamil Nadu India; 2 Department of Mechanical Engineering, Sri Sairam Engineering College, Chennai, Tamil Nadu India; 3 Department of Mechanical and Industrial Engineering, Manipal Institute of Technology, Manipal Academy of Higher Education, Manipal, India; 4 Department of Mechanical Engineering, Presidency University, Bengaluru, Karnataka India; 5 Department of Physics, Faculty of Engineering and Technology, JAIN (Deemed-to-be University), Bangalore, Karnataka India; 6 Department of Mechanical Engineering, Siksha ’O’ Anusandhan (Deemed to be University), Bhubaneswar, Odisha India; 7 Department of Mechatronics Engineering, Faculty of Engineering and Technology, Parul Institute of Technology, Parul University, Vadodara, Gujarat India; 8 Department of Physics, Saveetha School of Engineering, SIMATS, Saveetha University, Chennai, TamilNadu India

**Keywords:** Natural fibers, Plant waste, Sustainable development, Biological activities, Biotechnology, Chemistry, Materials science

## Abstract

The search for sustainable reinforcements in composite materials has increased interest in lignocellulosic fibers as eco-friendly alternatives to synthetic fibers. This study presents the first systematic evaluation of *Cosmos sulphureus* (CS) stem fibers, extracted via water retting, with emphasis on their antimicrobial, mechanical, morphological, and thermal properties. Antimicrobial activity: Agar diffusion assays against *Staphylococcus aureus* demonstrated concentration-dependent inhibition, with zones of 11 ± 0.5 mm (25 µg) and 17 ± 0.7 mm (50 µg), compared to 24 ± 0.6 mm for streptomycin (10 µg). Confocal laser scanning microscopy further confirmed biofilm disruption through degradation of the extracellular polymeric matrix. Mechanical performance: Tensile testing yielded a strength of 11.47 ± 0.42 MPa, elongation at break of 0.82%, and Young’s modulus of 1.4 GPa, indicating moderate strength but adequate stiffness for non-structural composite applications. Morphology: SEM micrographs revealed fibrillated surfaces, open lumens, and surface roughness, which are beneficial for polymer infiltration and fiber–matrix interfacial adhesion. Thermal stability: Thermogravimetric analysis showed an onset degradation at 346.62 °C, peak decomposition at 381.42 °C, and a residual char yield of 19.87% at 600 °C, underscoring appreciable thermal resilience. Although CS fibers exhibit lower tensile strength than bast fibers such as jute and flax, their adequate stiffness, intrinsic antimicrobial activity, and superior thermal stability establish them as eco-compatible reinforcements. These multifunctional attributes highlight their potential for non-structural applications, particularly in antimicrobial packaging, biomedical composites (subject to biocompatibility validation), and other sustainable engineering materials.

## Introduction

Natural fibers derived from agricultural products represent a sustainable and renewable resource with significant potential for human applications^1^. Agricultural residues, particularly fibrous stems from crops such as jute, banana, hemp, and sugarcane, are often discarded as waste after harvesting the primary yield. These by-products can be effectively valorised into high-value materials including textiles, ropes, packaging, polymer composites, and biomedical products^2^. The utilization of such fibers not only mitigates environmental concerns associated with residue burning or improper disposal but also promotes circular economy strategies by converting waste into functional products^3^. Structurally, these fibers are composed predominantly of cellulose, hemicellulose, and lignin, which impart desirable properties such as tensile strength, thermal stability, and biodegradability^4^. Their inherent biocompatibility and abundance further enhance their applicability in sectors ranging from engineering to healthcare, where they serve as reinforcements, absorbents, and bioactive scaffolds^5^. Thus, agricultural stem waste fibers provide a sustainable pathway for reducing reliance on synthetic materials while addressing global challenges in waste management and resource conservation^6^. Natural fibers derived from plant waste stems present distinct advantages for the development of sustainable green composites^7^. Their renewability and abundant availability reduce reliance on petroleum-derived synthetic reinforcements, thereby lowering the environmental footprint of composite production^8^. Owing to their low density, these fibers contribute to lightweight structures, while their favorable specific strength and stiffness enhance mechanical performance suitable for structural and semi-structural applications^9^. The high cellulose content promotes efficient stress transfer and interfacial adhesion with polymer matrices, whereas hemicellulose and lignin confer flexibility, thermal stability, and natural resistance to microbial degradation^10^. Beyond performance attributes, their intrinsic biodegradability and non-toxic nature support end-of-life recyclability and minimize ecological impact, aligning with circular economy and life-cycle sustainability frameworks^11^. Valorization of agricultural residues into functional fibers also mitigates the environmental hazards associated with residue burning and provides added economic value to agro-industrial systems^12^. Furthermore, these fibers are cost-effective, energy-efficient to process, and compatible with both thermosetting and thermoplastic polymers, enabling their integration into automotive, construction, packaging, and biomedical composites. Collectively, these characteristics establish plant waste-derived natural fibers as a critical enabler of eco-friendly, high-performance green composites for sustainable development^13^. Natural plant fibers exhibit intrinsic antibacterial activity due to the presence of bioactive constituents such as phenolic compounds, flavonoids, tannins, terpenoids, and lignin, which are retained within the fiber matrix during extraction^14^. These phytochemicals exert antimicrobial effects by disrupting microbial cell membranes, interfering with metabolic enzymes, and generating reactive oxygen species, ultimately inhibiting the proliferation of pathogenic bacteria^15^. Studies on fibers such as jute, banana, sisal, and hibiscus have reported measurable inhibition zones against both Gram-positive and Gram-negative strains, underscoring their efficacy as natural antimicrobial agents^16^. In contrast, synthetic fibers inherently lack antibacterial properties and generally require surface modification or the incorporation of chemical biocides, many of which pose environmental and health concerns^17^. Similarly, while animal-derived natural fibers such as wool or silk may offer limited antimicrobial resistance, they are comparatively expensive and less sustainable for large-scale applications^18^. Plant fibers derived from agricultural residues are abundant, cost-effective, and biodegradable, offering a unique combination of renewable availability, intrinsic antimicrobial function, and compatibility with composite matrices^19^. These attributes enhance product durability, improve hygienic performance in textiles and biomedical applications, and reduce reliance on environmentally hazardous chemical treatments. Consequently, plant fibers with inherent antibacterial properties represent a superior alternative to both synthetic and other natural fibers in the development of sustainable, multifunctional green composites^20^.

*Cosmos sulphureus* stem fibers, obtained from agricultural waste stems, exhibit physicochemical characteristics comparable to other natural bast fibers, making them promising reinforcements for sustainable composite applications. These fibers possess a relatively low density of 1.30–1.45 g/cm³, contributing to the development of lightweight composites when compared with conventional synthetic fibers such as glass. The fiber diameter ranges from 100 to 500 μm, depending on extraction conditions and plant maturity. Chemically, they are composed predominantly of cellulose (55–65 wt%), with hemicellulose and lignin contents of approximately 18–22 wt% and 10–15 wt%, respectively, providing structural stability and favorable interfacial bonding with polymer matrices^21^. The crystallinity index determined by X-ray diffraction is 60–65%, indicating a semi-crystalline structure that supports mechanical performance.

This study investigates the structural, functional, and performance attributes of *CS* stem fibers, valorised from agricultural waste, with the aim of advancing their use as sustainable reinforcements in biocomposites. The novelty of this work lies in the integrated evaluation of antibacterial, antibiofilm, mechanical, morphological, and thermal properties an approach not systematically reported in earlier studies. By coupling microbial resistance assays with detailed physicochemical characterization, this research provides a comprehensive understanding of the fibers’ multifunctional behavior, extending their potential beyond conventional reinforcement to advanced applications such as self-cleaning, biomedical, and eco-friendly packaging materials. Fibers were extracted using an environmentally benign water retting process and characterized through a suite of experimental methods: antibacterial efficacy via agar well diffusion assays, biofilm disruption by confocal laser scanning microscopy, tensile performance following ASTM standards, surface morphology using SEM, and thermal stability assessment through TGA. Collectively, this holistic framework highlights the promise of CS fibers as eco-compatible reinforcements for next-generation sustainable composite systems.

## Materials and experimental process

### Extraction of natural fibers from CS plant stem

**Fig. 1:**
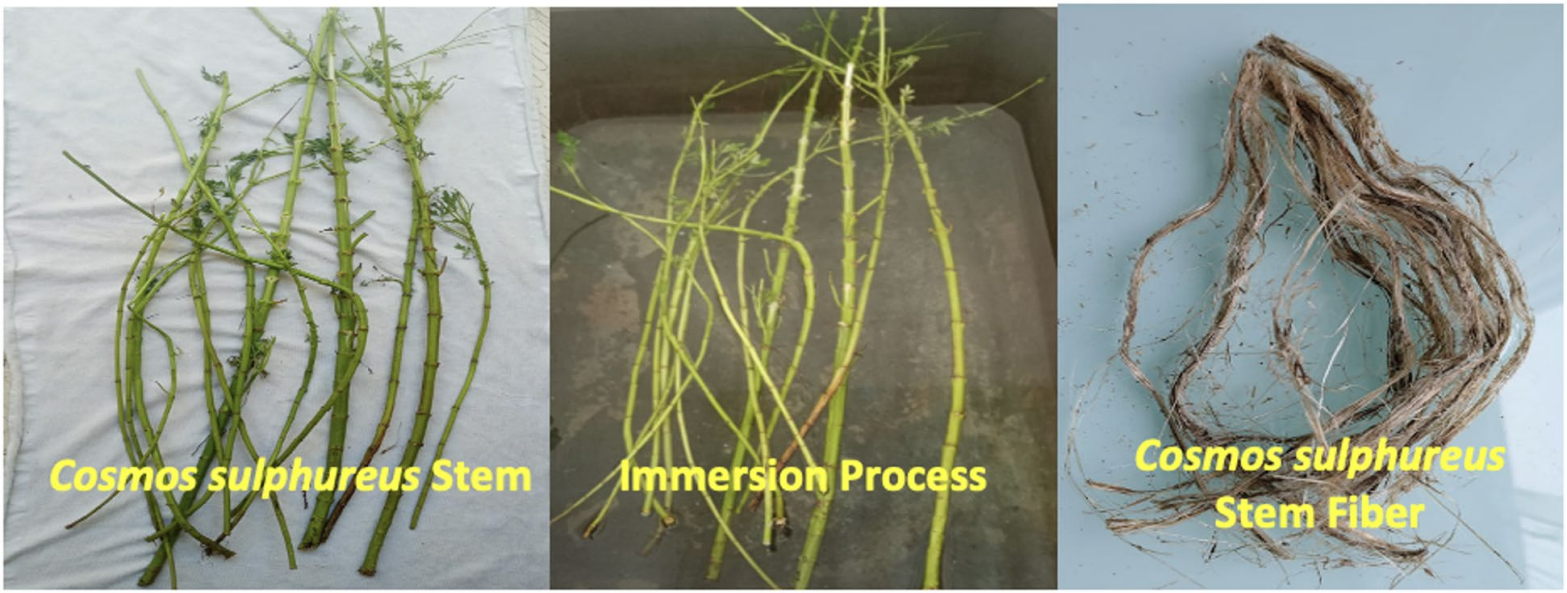
The fiber extraction process from CS plant stem.

The extraction of natural fibers from CS stems was conducted through a traditional water retting process, recognized as one of the most sustainable and environmentally benign methods for fiber separation. Mature stems of CS plants were harvested from cultivated fields in Polur, Tamil Nadu, India, and initially cleaned to remove leaves, branches, and surface debris. The cleaned stems were cut into manageable lengths (80–100 cm) and submerged in concrete retting tanks filled with fresh water, ensuring complete immersion. The retting period lasted between 18 and 22 days under local ambient conditions, where average daytime temperatures ranged from 28 to 34 °C, favouring microbial activity^22^. During this period, naturally occurring anaerobic and facultative anaerobic bacteria (such as *Clostridium* and *Bacillus* species) enzymatically degraded the pectin, hemicellulose, and other non-cellulosic materials binding the fibers to the woody core. The gradual enzymatic action led to the loosening of the fibrous bundles, enabling their separation without requiring chemical additives. Careful monitoring was performed to avoid under-retting, which results in poor fiber separation, or over-retting, which causes weakening of the fiber structure^23^. Once retting was completed, the fibers were manually extracted by gently stripping away the softened outer tissues of the stems. The liberated fibers were thoroughly washed in clean running water to remove decomposed residues, microbial deposits, and remaining non-cellulosic components. The fibers were then sun-dried for 72 h under the climatic conditions of Tamil Nadu to reduce moisture content to below 10%, thereby preventing microbial regrowth and ensuring long-term preservation. The dried fibers were stored in moisture-free conditions until further characterization. The use of a water retting process in Tamil Nadu not only ensures minimal environmental burden compared to chemical retting but also valorises agricultural residues into high-value functional fibers. Thus, the extraction of CS fibers through green retting provides a low-cost, sustainable, and scalable approach for producing renewable reinforcements suitable for green composites, textiles, packaging, and other eco-friendly engineering materials. Figure 1 reveals the fiber extraction process from CS plant stem.

### Testing process of CS fiber

**Fig. 2:**
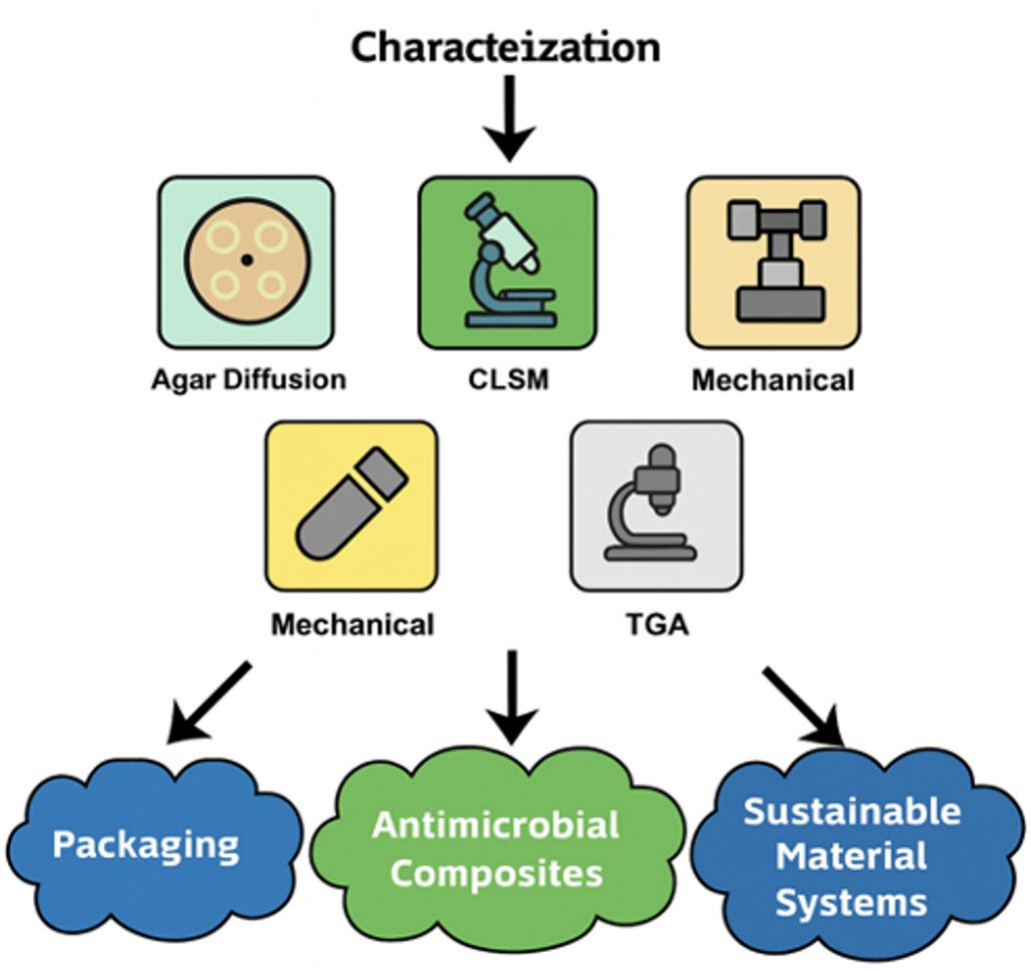
The schematic image for characterization of CS fiber.

The multifunctional performance of CS plant stem fibers was comprehensively evaluated through antibacterial, antibiofilm, mechanical, morphological, and thermal analyses. Antibacterial activity was assessed using the agar well diffusion method following Clinical and Laboratory Standards Institute (CLSI) guidelines, with *Staphylococcus aureus* selected as the model Gram-positive pathogen. Nutrient agar plates were seeded with a standardized bacterial suspension (10⁶ CFU/mL), and 6 mm diameter wells were loaded with fiber extract solutions at concentrations of 25 and 50 µg/mL. *Streptomycin* (10 µg) was included as the positive control, while sterile distilled water served as the negative control. Plates were incubated at 37 °C for 24 h, after which inhibition zones were measured, revealing concentration-dependent antibacterial activity^24^. Antibiofilm efficacy was further examined using confocal laser scanning microscopy (CLSM, Zeiss LSM 880, Carl Zeiss, Germany). *Staphylococcus aureus* biofilms treated with fiber extracts were stained with SYTO9 and propidium iodide (Live/Dead BacLight kit), and imaged under excitation wavelengths of 488 and 561 nm. The CLSM micrographs confirmed significant disruption of biofilm architecture and reduced bacterial viability in treated samples^25^. Mechanical properties were determined by single-fiber tensile testing in accordance with ASTM D3822-14. Fibers with a gauge length of 50 mm were mounted on paper frames and tested at a crosshead speed of 2 mm/min using a Universal Testing Machine (Instron 3369, USA) fitted with a 10 N load cell. Prior to testing, samples were conditioned for 48 h at 65% relative humidity and 27 ± 2 °C. Tensile strength, Young’s modulus, and elongation at break were calculated from the stress–strain curves. Surface morphology and fracture characteristics were examined using scanning electron microscopy (SEM, JEOL JSM-IT300, Japan). Fibers were sputter-coated with a 10 nm gold layer using a Quorum Q150R ES coater, and imaging was conducted at voltage of 15 kV, which revealed well-defined fibrillar structures, surface roughness, and fracture features indicative of fiber–matrix compatibility^26^. Thermal stability was evaluated by thermogravimetric analysis (TGA, TA Instruments Q500, USA). A 10 mg of fiber specimens were heated from 30 °C to 600 °C at a heating rate of 10 °C/min under nitrogen flow. The thermal degradation profile showed an initial weight loss associated with moisture evaporation, followed by major decomposition attributed to hemicellulose and cellulose breakdown, with lignin contributing to residual char stability. Onset degradation temperatures, maximum decomposition rates, and final char yields were recorded, confirming the fibers’ thermal endurance for composite reinforcement^27^. Figure 2 shows the schematic image for characterization of CS fiber.

## Results and discussion

### Antibacterial activities of CS fiber

**Fig. 3:**
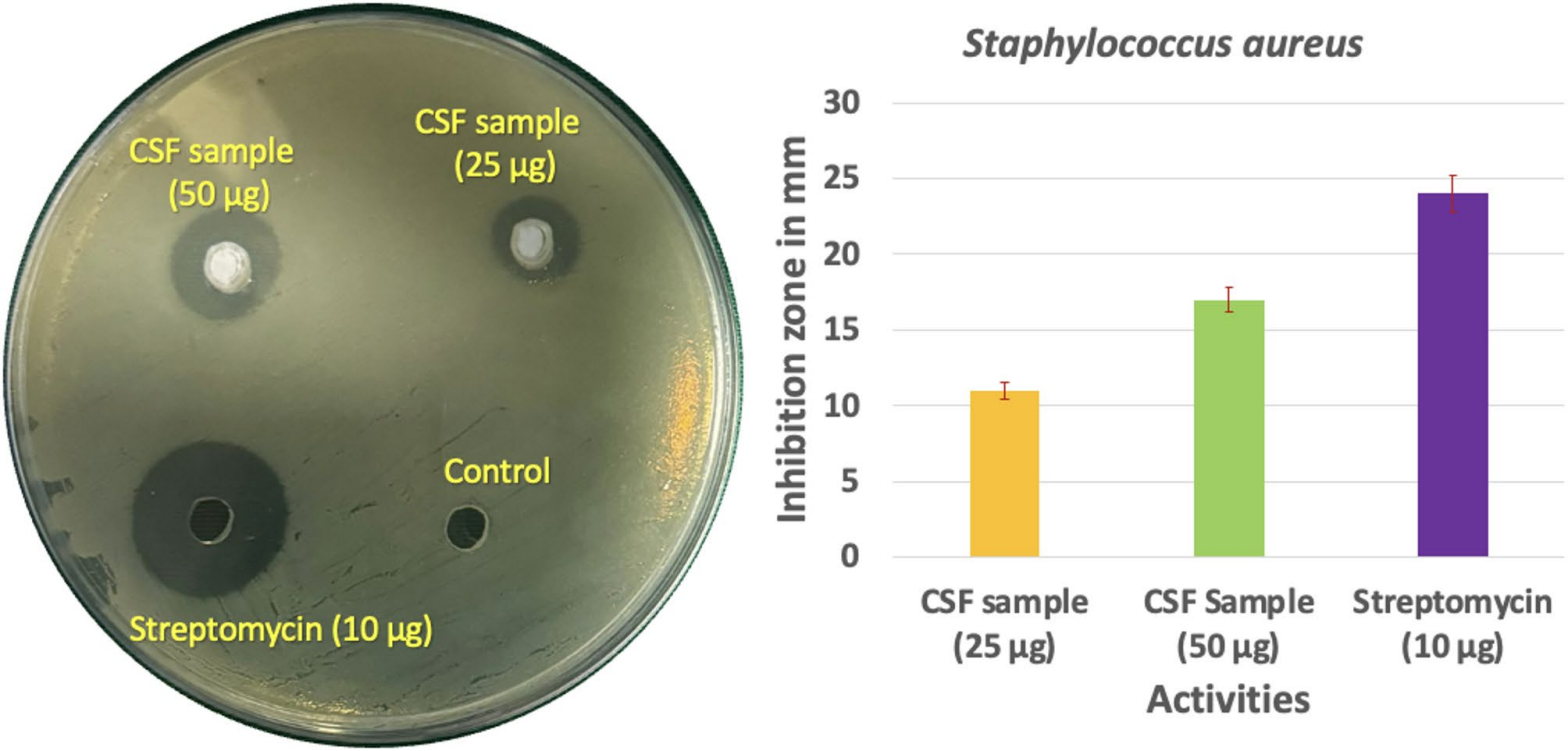
The antibacterial inhibition zone of CS fiber.

The antibacterial activity of *Cosmos sulphureus* fiber (CSF) extracts against *Staphylococcus aureus* was quantitatively assessed using the agar well diffusion method, and the results demonstrated a concentration-dependent response. At a lower concentration of 25 µg, CSF produced an inhibition zone of 11 ± 0.5 mm, while at a higher concentration of 50 µg, the inhibition zone increased to 17 ± 0.7 mm. In comparison, the standard antibiotic streptomycin (10 µg) exhibited the maximum inhibition zone of 24 ± 0.6 mm. These results indicate that CSF extracts at 25 µg displayed 54.1% lower antibacterial efficacy relative to streptomycin, while the 50 µg extract showed 29.1% lower inhibition. The improvement from 11 mm to 17 mm represents approximately a 54.5% enhancement in antibacterial activity, emphasizing the role of increasing concentration in enhancing bioactivity. Figure 3 shows the antibacterial inhibition zone of CS fiber.

The antibacterial mechanism of CS fibers is attributed to phytochemical constituents such as phenolic acids, flavonoids, tannins, and lignin derivatives that are retained in the lignocellulosic matrix. These compounds act synergistically to disrupt bacterial cell wall integrity, induce oxidative stress, increase membrane permeability, and cause leakage of cytoplasmic contents. Functional groups such as hydroxyl and carbonyl moieties can bind to bacterial proteins and enzymes, resulting in inactivation of vital metabolic processes, while flavonoids can chelate essential metal ions needed for microbial proliferation^28^. Unlike streptomycin, which specifically inhibits ribosomal protein synthesis, the antibacterial effect of CSF extracts is broad-spectrum and multi-targeted, thereby reducing the likelihood of rapid bacterial resistance. When compared with other natural fibers, CSF demonstrates a comparable antibacterial effect. For example, jute fibers have been reported to exhibit inhibition zones in the range of 9–12 mm against *S. aureus* due to the presence of lignin-derived phenolics^29^. Similarly, banana fibers showed inhibition zones of 13–16 mm when tested against Gram-positive bacteria, linked to their higher polyphenol content^30^. Hibiscus cannabinus fibers exhibited zones of 10–14 mm^31^, while flax fibers demonstrated moderate antibacterial activity, with inhibition zones averaging around 12–15 mm^32^. The inhibition zone of CSF at 50 µg (17 mm) is at the higher end of this range, suggesting superior antibacterial potential relative to several commonly studied plant fibers. These variations across fiber types can be attributed to differences in cellulose crystallinity, lignin content, and the concentration of secondary metabolites responsible for antimicrobial action. The observed differences between CSF extracts and streptomycin are largely due to the purity and specificity of action of the antibiotic compared to the mixed phytochemical profile of plant fibers. Although streptomycin achieved a higher inhibition zone, natural fibers such as CSF provide the added advantages of sustainability, biodegradability, and inherent multifunctionality. Moreover, unlike antibiotics that target a specific molecular pathway, CSF’s phytochemical-mediated antibacterial activity acts on multiple targets simultaneously, which is beneficial for applications requiring long-term antibacterial resistance. Finally, *CS* stem fibers exhibit antibacterial activity comparable to or higher than several other natural fibers, though still below the potency of synthetic antibiotics. Their concentration-dependent response, broad-spectrum mechanism, and renewable nature make them highly promising for incorporation into eco-friendly composites, biomedical devices, and antimicrobial packaging materials, offering a balance between functional performance and sustainability. The concentrations of 25 µg and 50 µg were selected based on preliminary range-finding assays, which revealed reproducible inhibition zones beginning at 25 µg and a clear enhancement at 50 µg without diffusion artifacts. These values also fall within commonly reported ranges (10–100 µg) for agar diffusion screening of plant-derived materials. It should be noted that these inhibition-zone results serve as preliminary indicators of antibacterial potential, while definitive MIC and MBC values should be established through standardized broth microdilution methods as recommended by CLSI/EUCAST protocols.

### Biofilm analysis of CS fiber

**Fig. 4:**
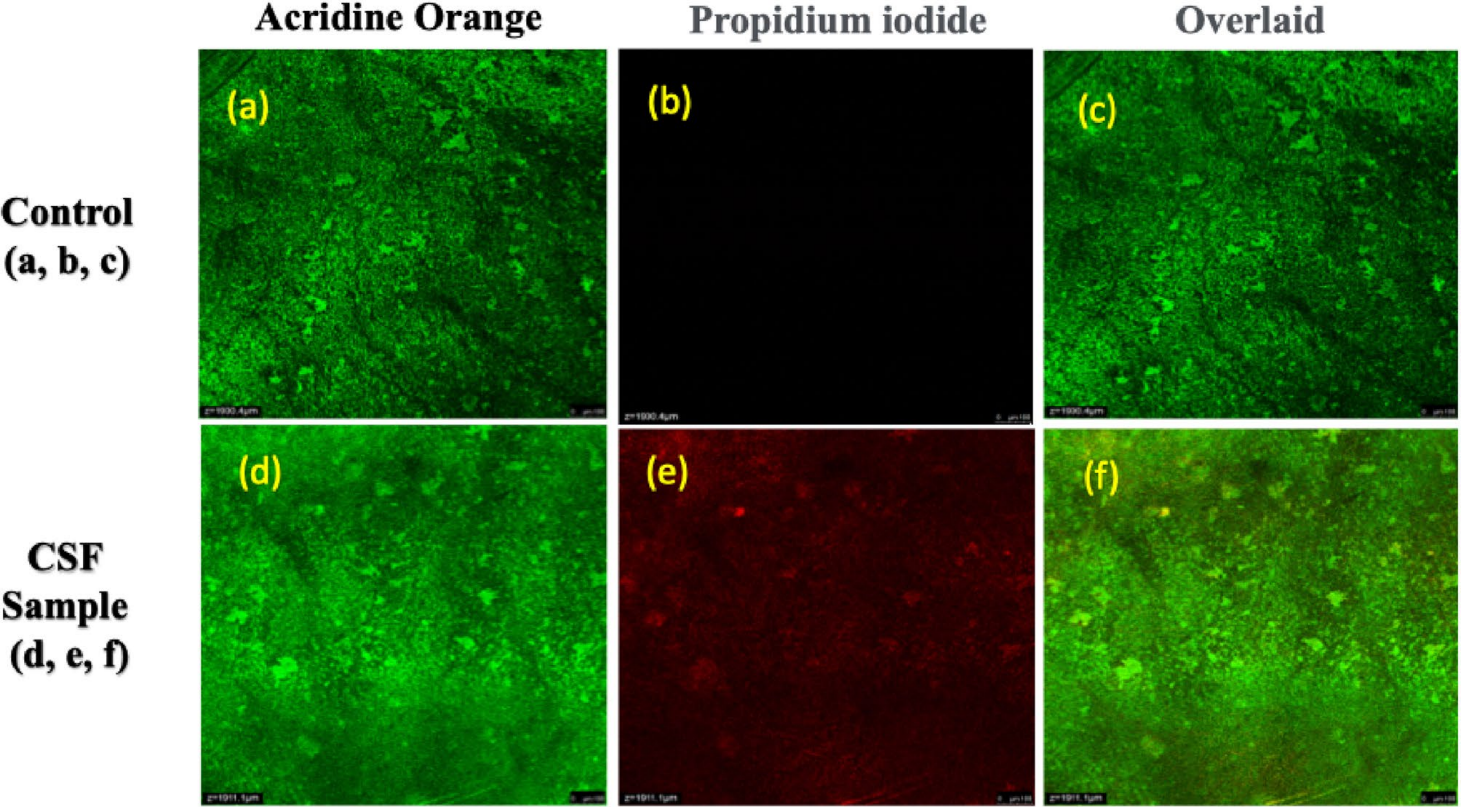
The anti-biofilm images of CS fiber.

The antibiofilm activity of CSF extracts against *Staphylococcus aureus* was assessed through fluorescence staining using acridine orange (AO) and propidium iodide (PI) coupled with CLSM. In the untreated control biofilms, dense and uniform green fluorescence was observed following AO staining, indicative of metabolically active cells with intact cell membranes. The absence of significant red fluorescence under PI staining further confirmed high bacterial viability within the biofilm community, while the merged images revealed compact and well-organized biofilm architecture, representative of mature biofilm formation. In contrast, biofilms exposed to CSF extracts exhibited a marked reduction in green fluorescence intensity, with extensive red fluorescence observed in the PI channel. This fluorescence shift from green to red strongly suggests membrane compromise, cell death, and significant disruption of bacterial integrity. The merged CLSM images of treated biofilms displayed heterogeneous fluorescence patterns, reflecting a mixed population of damaged and dead bacterial cells, along with visibly altered and fragmented biofilm structures compared to the dense clustering in untreated controls. The disruption of biofilms by CSF extracts can be mechanistically explained by the synergistic action of phytochemical constituents, particularly phenolic acids, flavonoids, and tannins, which interfere with bacterial survival pathways. These compounds destabilize the extracellular polymeric substance (EPS) matrix through oxidative interactions and hydrogen bonding, reducing the mechanical stability of biofilms. By weakening EPS integrity^33^, CSF treatment limits bacterial adhesion and aggregation, thereby inhibiting biofilm maturation. Furthermore, phenolic groups can bind to bacterial proteins and enzymes, leading to denaturation and impairment of essential metabolic functions. Flavonoids are also known to inhibit quorum sensing pathways, disrupting cell-to-cell communication that is critical for biofilm initiation and persistence. The combined effect is a reduction in both bacterial viability and the structural robustness of biofilms. Figure 4 shows the anti-biofilm images of CS fiber.

Quantitatively, the treatment led to a significant reduction in viable cells compared to the control. While control biofilms displayed nearly complete green fluorescence coverage, CSF treatment resulted in a substantial decrease in green signal intensity with a corresponding increase in red-stained dead cells. Based on image analysis, the live-to-dead cell ratio decreased markedly in treated biofilms, reflecting a clear decline in bacterial survival within the biofilm matrix. This outcome demonstrates that CSF extracts not only exhibit antibacterial effects but also possess antibiofilm activity, an essential property considering that biofilms are inherently more resistant to conventional antibiotics than planktonic bacteria. In the broader context of natural fibers, this antibiofilm activity of CSF aligns with findings from other lignocellulosic fibers such as jute and banana, which also possess moderate antibiofilm potential due to their phytochemical composition. However, the higher red fluorescence intensity observed in CSF-treated biofilms compared to those reported for other fibers suggests a relatively stronger disruption capability, potentially linked to its higher concentration of secondary metabolites. While synthetic antibiotics like streptomycin remain more potent in absolute terms, their action is often single-targeted, whereas CSF exerts a multi-target mechanism that reduces the risk of bacterial resistance development. Therefore, the fluorescence-based antibiofilm analysis demonstrates that CS fibers not only inhibit planktonic bacterial growth but also effectively disrupt biofilm formation by damaging the EPS matrix, compromising membrane integrity, and interfering with bacterial communication systems. This dual antibacterial–antibiofilm activity underscores the value of CSF as a sustainable biomaterial with strong potential for integration into biomedical devices, wound healing dressings, and antimicrobial packaging materials, where long-term resistance to microbial colonization is essential. While CLSM images confirmed disruption of the extracellular polymeric matrix, quantitative metrics such as biomass reduction, biofilm thickness, and fluorescence intensity were not measured in this study. This is acknowledged as a limitation, and future work will incorporate software-based image analysis (e.g., COMSTAT, ImageJ) to provide statistically validated biofilm inhibition data.

### Tensile strength of CS fiber

**Fig. 5:**
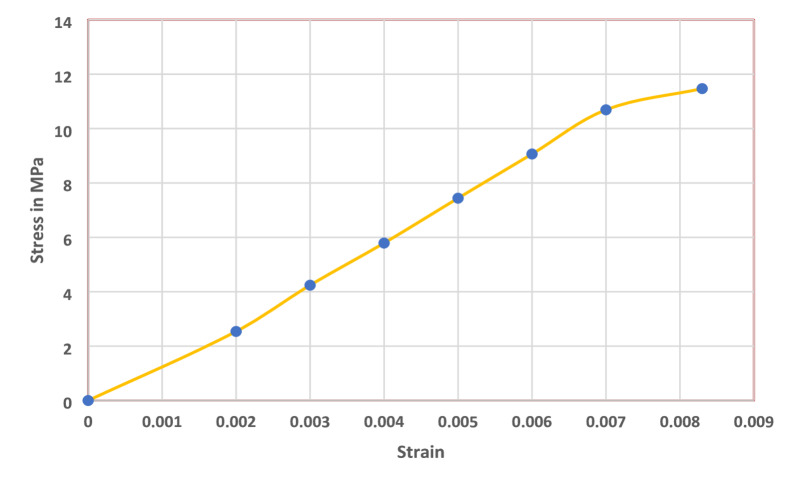
The stress vs. strain curve of *CS* fiber.

The tensile performance of CS stem fibers was evaluated to establish their mechanical characteristics for potential reinforcement in sustainable composites. The average tensile strength of CS stem fibers was 11.47 ± 0.42 MPa (*n* = 3) at a strain of 0.0082 (0.82%), with a Young’s modulus of ~ 1.4 GPa, demonstrating adequate stiffness despite lower strength compared to bast fibers. Compared to other fibers, CS fibers show much weaker tensile properties: jute demonstrates tensile strengths between 20 and 30 MPa with moduli of 1–3 GPa, flax ranges from 15 to 29 MPa with moduli of 1–2 GPa, banana fibers exhibit 18–34 MPa strength with 2–6 GPa modulus, and kenaf shows tensile strengths of 12.95–29.3 MPa with moduli of 1.4–2 GPa^34^. The much lower strength and stiffness of *CS* fibers are attributable to their relatively lower cellulose crystallinity and higher hemicellulose–lignin fractions, which compromise fibril alignment and reduce load-bearing capacity. Figure 5 shows the stress vs. strain curve of CS fiber.

Although the stress–strain profile suggests a gradual rise in strain before fracture, this does not indicate true ductility. Natural fibers are quasi-brittle materials, and their failure occurs abruptly once the maximum load is reached. The curvature in the stress–strain curve is mainly due to microstructural mechanisms such as microfibril reorientation, fibril slippage, and progressive crack propagation in the fiber wall, which allow limited strain accommodation without permanent plastic deformation. In contrast, bast fibers such as flax and jute fail at much lower strains (1.5–3%), while CS shows a somewhat comparable elongation of 0.82%, albeit at a much lower stress level. This behavior suggests that while CS fibers cannot compete with established bast fibers in high-strength applications, their moderate stiffness, quasi-brittle fracture mode, and renewable origin make them suitable for non-structural biocomposites, biodegradable packaging, and insulation products where flexibility, sustainability, and functional properties such as antibacterial activity are prioritized over high mechanical strength^35^. Additionally, surface treatments, chemical modifications, or hybridization with stronger fibers may enhance their mechanical performance and broaden their applicability in eco-friendly engineering materials. The elongation at break of CS fibers was found to be 0.82%, reflecting their brittle nature. This low ductility may limit the flexibility and toughness of biocomposites reinforced solely with CS fibers, potentially affecting impact resistance and energy absorption. To address this limitation, strategies such as hybridization with more ductile fibers, incorporation of elastomeric modifiers, or surface treatments to enhance fiber–matrix adhesion can be employed, thereby improving the overall toughness and practical usability of CS fiber–based composites.

### SEM analysis of CS fiber

**Fig. 6:**
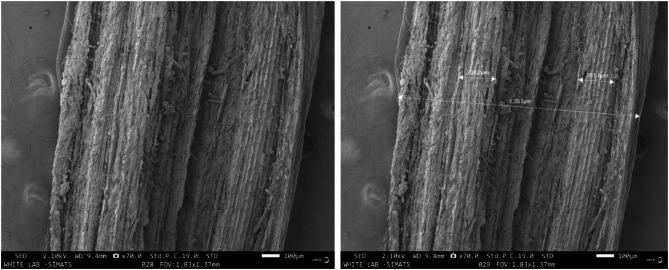
The SEM morphology of CS fiber.

The SEM microstructural examination of raw CS stem fibers revealed a highly heterogeneous and anisotropic architecture typical of lignocellulosic reinforcements. The fibers appeared as aggregated bundles exhibiting longitudinal fibrillar alignment interspersed with striated ridges, grooves, and discontinuities, reflecting their native hierarchical arrangement. The external surface displayed pronounced topographical irregularities, with roughened contours, micro-voids, and particulate residues, indicative of residual hemicellulose, lignin, and pectic substances incompletely removed during the retting and manual decortication process. Fiber diameters exhibited considerable heterogeneity, with individual fibrils ranging between 200 and 350 μm and bundle widths extending to 1350 μm, suggesting non-uniform fibril packing density and structural inhomogeneity. These intrinsic discontinuities, coupled with void formation, serve as potential stress localization sites under tensile loading, predisposing the fibers to premature microcrack initiation and brittle fracture. To complement the qualitative observations, fiber diameters were statistically analyzed, revealing an average diameter of 261 ± 42 μm across 10 measured fibrils. This distribution confirms the inherent heterogeneity of CS fibers and supports the SEM-based observation of non-uniform fibril packing. Reporting the mean ± SD provides a quantitative basis for correlating fiber morphology with mechanical behavior and highlights the variability that may influence composite performance. Conversely, the presence of such asperities and surface coarseness may be advantageous in composite systems, where they provide enhanced interfacial anchoring and frictional interlocking with the polymer matrix, improving load transfer efficiency despite the fibers’ relatively moderate intrinsic strength. Figure 6 shows the SEM morphology of CS fiber.

When juxtaposed with other natural fibers, the morphology of CS exhibits both parallels and divergences. Henequen fibers present with more polygonal fibril bundling and comparatively smoother exteriors, though residual non-cellulosic matrices necessitate alkali treatments for interfacial optimization^36^. Banana fibers, by contrast, are characterized by coarser textures, luminal porosity, and fibrillar delamination, mirroring the morphological irregularities of CS and conferring similar variability in mechanical reliability^37^. Kenaf fibers generally exhibit tighter fibrillar alignment and reduced extraneous impurities, resulting in comparatively greater mechanical consistency^38^, whereas flax fibers are renowned for their refined microfibrillar orientation, reduced surface defects, and superior crystallinity, which collectively underpin their elevated tensile strength and stiffness. In this comparative context, CS fibers occupy an intermediate position: their rugged surface morphology and heterogeneous fibril geometry yield lower intrinsic mechanical properties (tensile strength ~ 11.47 MPa; modulus ~ 1.4 GPa) than flax or kenaf, yet provide a naturally roughened interface conducive to polymer adhesion, similar to banana and jute. Therefore, SEM observations affirm that CS fibers possess a distinctively coarse, irregular, and impurity-laden morphology, which, while compromising structural uniformity and strength, enhances their compatibility in biocomposites applications through improved mechanical interlocking and wettability. Their morphological signature thus situates them closer to banana and jute fibers rather than refined bast fibers like flax, aligning them with applications where eco-efficiency, biodegradability, and interfacial bonding are prioritized over high structural strength.

### Thermal stability of CS fiber

**Fig. 7:**
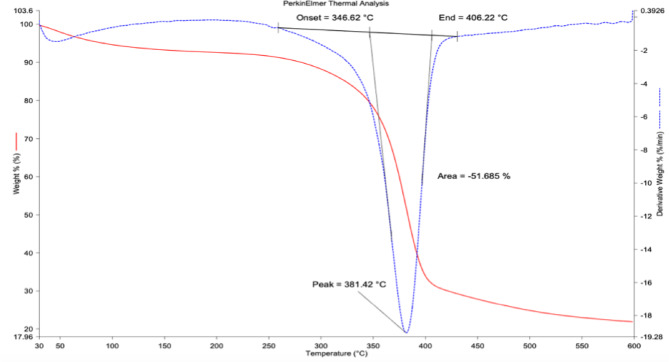
The TGA curve of CS fiber.

The thermogravimetric (TGA) and derivative thermogravimetric (DTG) profiles of CS fiber demonstrate a characteristic multi-stage thermal decomposition behavior, reflective of its heterogeneous lignocellulosic constitution. The first degradation step, observed below 119 °C, is attributed to the desorption of physically adsorbed moisture and the volatilization of low-molecular-weight extractives, contributing to a marginal weight reduction of 3–5%. The primary decomposition stage initiates at the onset temperature of 346.62 °C, signifying the thermal destabilization of hemicellulose fractions and the initial scission of amorphous cellulose chains. The DTG curve exhibits a pronounced degradation peak at 381.42 °C, corresponding to the extensive depolymerization of cellulose via cleavage of β−1,4-glycosidic linkages and the evolution of volatile pyrolytic products such as levoglucosan, furans, and an hydro sugars^39^. This event is thermodynamically the most critical, resulting in a substantial mass reduction of ~ 51.69%, and is accompanied by a sharp exothermic manifestation indicative of chain fragmentation and volatilization processes. The decomposition is completed at the offset temperature of 406.22 °C, beyond which cellulose degradation tapers off. Figure 7 shows the TGA curve of CS fiber.

Subsequent to cellulose pyrolysis, the thermal decomposition is governed predominantly by the progressive degradation of lignin, a complex polyaromatic polymer. Unlike cellulose and hemicellulose, lignin degrades over a broad temperature interval (200–600 °C) due to its heterogeneity and the presence of methoxy-substituted phenylpropane units. This phase involves sequential reactions including demethoxylation, side-chain scission, cleavage of ether linkages, and eventual aromatization, culminating in the formation of a thermally resilient carbonaceous char^40^. The residual mass (19.87% at 600 °C) signifies the high char yield of CS fiber, which is advantageous for thermal insulation, as the aromatic structures confer dimensional stability and resist complete volatilization. From a mechanistic perspective, the high onset temperature (346.62 °C) and elevated DTG peak (381.42 °C) compared to fibers such as jute (320–340 °C)^41^, flax (~ 330 °C), and sisal (~ 325 °C)^42^ underscore the superior structural integrity and higher cellulose crystallinity of CS fiber. The delayed decomposition reflects strong intermolecular hydrogen bonding and the presence of thermally stable lignin moieties that act as natural stabilizers. The significant char residue further suggests a high lignin-to-cellulose ratio and mineral content, which enhances dehydration pathways and catalyses aromatic condensation during pyrolysis. This thermal resistance not only validates the fiber’s potential in high-temperature polymer processing (below 280–300 °C) but also positions it suggesting as a reinforcement for thermoplastic and thermoset composites for dimensional stability applications. The formation of a stable carbonaceous char layer can act as an insulating barrier, reducing heat release, slowing mass transfer of volatile products, and limiting oxygen diffusion to the combustion zone. Such properties are advantageous for applications in packaging and automotive composites, where thermal resistance and fire safety are important performance criteria. While the inherent char yield of CS fibers alone may not achieve full flame-retardant standards, it provides a valuable baseline that could be further optimized through fiber–matrix hybridization or the incorporation of flame-retardant additives.

## Conclusion

This study demonstrated that *Cosmos sulphureus* stem fibers, extracted through green retting, exhibit multifunctional characteristics that make them promising as sustainable reinforcements in biocomposites. The fibers showed antibacterial and antibiofilm activity against *Staphylococcus aureus*, highlighting their potential for use in hygienic and antimicrobial composite systems. Mechanical testing confirmed moderate tensile strength with intrinsic brittleness, supported by SEM evidence of a surface morphology favorable for interfacial adhesion. Thermogravimetric analysis revealed appreciable thermal stability with significant char residue formation, indicating resistance to thermal degradation. Collectively, these attributes establish CS fibers as eco-compatible reinforcements that combine antimicrobial efficacy, mechanical reliability, and thermal resilience. While their multifunctional performance suggests scope for packaging and antimicrobial applications, prospective biomedical use will require further validation through standardized biocompatibility and cytotoxicity testing. This work therefore advances the valorization of agricultural residues into sustainable, high-value engineering materials.

## Data Availability

The datasets used and/or analysed during the current study available from the corresponding author on reasonable request.
